# The Impact of Sugar Cane–Burning Emissions on the Respiratory System of Children and the Elderly

**DOI:** 10.1289/ehp.8485

**Published:** 2006-01-13

**Authors:** José E.D. Cançado, Paulo H.N. Saldiva, Luiz A.A. Pereira, Luciene B.L.S. Lara, Paulo Artaxo, Luiz A. Martinelli, Marcos A. Arbex, Antonella Zanobetti, Alfesio L.F. Braga

**Affiliations:** 1 Environmental Epidemiology Study Group, Laboratory of Experimental Air Pollution, University of São Paulo Medical School, São Paulo, Brazil; 2 Community Health Post-graduation Program, Catholic University of Santos, Santos, Brazil; 3 Center for Nuclear Energy in Agriculture, University of São Paulo, Piracicaba, Brazil; 4 Physics Institute, University of São Paulo, São Paulo, Brazil; 5 Pulmonary Physiopathology and Air Pollution Research Group, Internal Medicine Department, Federal University of São Paulo Medical School, São Paulo, Brazil; 6 Exposure Epidemiology and Risk Program, Department of Environmental Health, Harvard School of Public Health, Boston, Massachusetts, USA; 7 Environmental Pediatrics Program, University of Santo Amaro Medical School, São Paulo, Brazil

**Keywords:** air pollution, biomass burning, children, elderly people, health effects, Poisson regression, respiratory diseases, time series

## Abstract

We analyzed the influence of emissions from burning sugar cane on the respiratory system during almost 1 year in the city of Piracicaba in southeast Brazil. From April 1997 through March 1998, samples of inhalable particles were collected, separated into fine and coarse particulate mode, and analyzed for black carbon and tracer elements. At the same time, we examined daily records of children (< 13 years of age) and elderly people (> 64 years of age) admitted to the hospital because of respiratory diseases. Generalized linear models were adopted with natural cubic splines to control for season and linear terms to control for weather. Analyses were carried out for the entire period, as well as for burning and nonburning periods. Additional models were built using three factors obtained from factor analysis instead of particles or tracer elements. Increases of 10.2 μg/m^3^ in particles ≥ 2.5 μm/m^3^ aerodynamic diameter (PM_2.5_) and 42.9 μg/m^3^ in PM_10_ were associated with increases of 21.4% [95% confidence interval (CI), 4.3–38.5] and 31.03% (95% CI, 1.25–60.21) in child and elderly respiratory hospital admissions, respectively. When we compared periods, the effects during the burning period were much higher than the effects during nonburning period. Elements generated from sugar cane burning (factor 1) were those most associated with both child and elderly respiratory admissions. Our results show the adverse impact of sugar cane burning emissions on the health of the population, reinforcing the need for public efforts to reduce and eventually eliminate this source of air pollution.

The impact of biomass and fossil fuel burning is felt throughout the world. Although studies have documented the impact of fossil fuel air pollution on health (Braga et al. 2001; Hoek and Brunekreef 1994; Hoek et al. 1997; Laden et al. 2000; Lin et al. 1999; Pope et al. 1995; Saldiva et al. 1994; Schwartz and Dockery 1992; Schwartz et al. 2001), there is a scarcity of information on biomass burning [Arbex et al. 2000; Brauer and Hisham-hashim 1998; Long et al. 1998; Phonboon et al. 1999; World Health Organization (WHO) 1998]. Most biomass burning is carried out in developing countries and is done to clear land for shifting cultivation, to convert forests to agricultural or pastoral lands, and to remove dry vegetation to promote agricultural productivity. Burning of agricultural wastes in the field, such as sugar cane and stalks from grain crops, is another important type of biomass burning (Artaxo et al. 1999; Crutzen and Andreae 1990).

Biomass burning emissions represent an important global source of particles and gases to the atmosphere, especially in the tropics where biomass burning is widespread. Annually, 7,500–8,600 Tg (teragrams) of dry material is emitted to the atmosphere around the world through the process of burning. About 43% of this dry material is derived from savannah burning, 23% from the burning of agricultural waste, 18% from rainforest burning, and 16% from wood burning for fuel (Levine et al. 1995). Biomass burning emits large amounts of carbon and particulate matter (PM). Estimates show that the annual carbon and PM released to the atmosphere because of biomass fires in the tropics is around 2,000–4,500 Tg and 36–154 Tg, respectively (Crutzen and Andreae 1990).

Brazil plays an important role in biomass burning emissions. Most of the fires in Brazil occur during the dry season, from May through October. An especially critical area in the country is the Amazon region, where every year approximately 17,000 km^2^ of tropical forests are cut down and most of them burned. Until now, of the total Amazon area (5.5 million km^2^), 14% has been deforested (Instituto Nacional de Pesquisas Espaciais 2002).

In São Paulo State, located in the southeastern region of Brazil, most of the fires are generated in agricultural fields, especially sugar cane crops, in which 20 tons/ha are burned every year to facilitate harvesting. Sugar cane fires also have significant effects on the composition and acidity of rainwater over large areas of southeastern Brazil because of the emissions of aerosol and trace gases (Lara et al. 2001). Aerosols can also cause injury to human health. Small PM, particularly those < 2.5 μm in aerodynamic diameter (PM_2.5_), appear to have the greatest potential for damaging health because they can penetrate deep into the lungs and reach the lower respiratory system (American Thoracic Society 1996). Despite its importance, little information about the aerosol particles emitted from sugar cane burning is available (Lara et al. 2005; Martinelli et al. 2002).

Within this scenario, the Piracicaba region, located in São Paulo State, is especially interesting because the atmosphere of this region receives emissions not only from industrial and urban sources but also and primarily from sugar cane burning (Lara et al. 2001). In this study, we investigated the influences of PM generated from sugar cane burning on respiratory hospital admissions of children and the elderly.

## Materials and Methods

### Study area and sampling.

**Figure 1:**
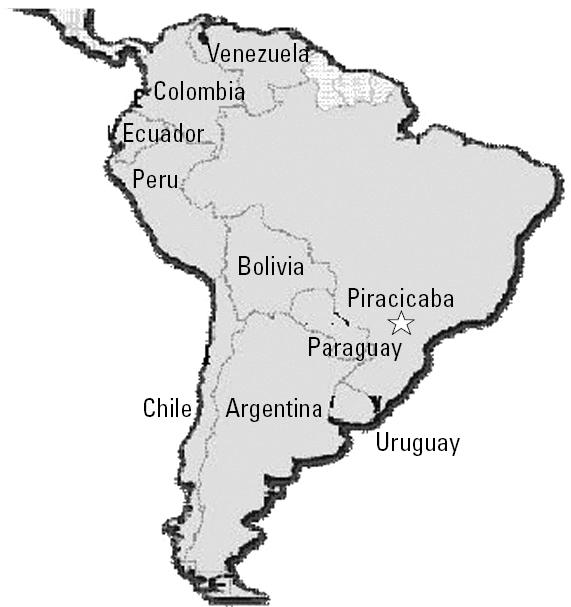
Location of the city of Piracicaba (sampling site) in São Paulo State, southeastern Brazil.

The city of Piracicaba is located in the western part of the Piracicaba River basin, in São Paulo State, and has a population of approximately 320,000 inhabitants and a population density of 242 inhabitants/km^2^ (Figure 1). The land use in this area is dominated by sugar cane plantations (80%), followed by pastures (11%), urban areas (6%), and forests (3%) (Centro de Energia Nuclear para a Agronomia 1997). Sugar cane is burned every year from May through October, during the dry season, whereas the wet season extends from November through April.

From April 1997 through March 1998, samples of inhalable particles (PM_10_) were taken from four stacked filter units (SFU) in two separated size fractions: fine PM (PM_2.5_) and coarse PM (2.5 < particle diameter < 10 μm). Each filter unit was a 47-mm diameter Nuclepore polycarbonate filter (Whatman International Ltd., Kent, UK) and was retrieved every 72 hr. Samplings were obtained at the meteorologic station of the Escola Superior de Agricultura Luiz de Queiroz, which is located about 4 km from downtown Piracicaba and < 1 km from the nearest sugar cane plantation. The SFU inlets were located 3 m above the ground to minimize direct influences of local resuspended soil dust. The dominant wind direction was from the sugar cane plantations to the sampling station. Gravimetric mass and black carbon (BC) were measured in the Nuclepore filters. BC was measured by a reflectance technique according to the method developed by Reid et al. (1998).

Elemental composition was measured by particle-induced X-ray emission (PIXE) (Johansson and Campbell 1998), a multi-elemental technique that allows the identification of 21 elements (aluminum, silicon, phosphorus, sulfur, chlorine, potassium, calcium, titanium, vanadium, chromium, manganese, iron, nickel, copper, zinc, selenium, bromine, rubidium, strontium, zirconium, lead). PIXE detection limits are about 1 ng/m^3^ for elements with Z > 20 and about 10 ng/m^3^ for elements from sodium to K. Details on PIXE technique and aerosol sampling have been reported by Artaxo et al. (1999) and Yamasoe et al. (2000).

To investigate the respiratory health effects on the Piracicaba population, we obtained daily records of hospital admissions due to respiratory diseases [categorized according to *International Classification of Diseases,* 9th revision, (ICD-9; WHO 1975) codes 460–519 and *International Classification of Diseases*, 10th revision (ICD-10; WHO 1994) codes J00–J99] for children (< 13 years of age) and the elderly (> 64 years of age) from the government health agency (DATASUS; Brasilia, Brazil) from April 1997 to March 1998. The Agricultural School of São Paulo University (Piracicaba, Brazil) provided daily records of minimum temperature and relative humidity.

### Statistical analysis of air pollutant health effects.

Counts of daily respiratory hospital admissions for children and elderly were modeled separately for the entire period in Poisson regressions. We used the generalized linear model (McCullagh and Nelder 1989) with natural cubic splines (Green and Silverman 1994) to control for season. Splines were used to account for the nonlinear dependence of the hospital admissions on that covariate.

The function of time was used to remove the basic seasonal pattern (and long-term trend) from the data. If each admission were an independent event, we would expect no serial correlation in the data. Seasonal patterns for each end point (child and elderly respiratory hospital admissions) were modeled. Because we assumed that seasonal patterns would vary according to the adopted end point, end point–specific time smoothing parameters were used.

It was not necessary to incorporate autoregressive terms (Brumback et al. 2000) in the models because autocorrelation plots showed there were no remaining serial correlations in the residual. Indicators for day of the week were included in order to control for short-term trends.

Respiratory diseases present an almost linear relationship with weather. Therefore, linear terms for temperature and relative humidity were adopted. To reduce sensitivity to outliers in the dependent variable, we used robust regression (M-estimation). Three-day moving averages of BC, PM, and their main tracer elements were used to estimate the effects on respiratory morbidity.

For each age group, using the coefficients of the pollutants that presented adverse effects on respiratory hospital admissions for the entire period, we estimated the effects in two different periods: burning (May through October) and nonburning (November through April).

Additionally, because of the large number of elemental components, specific rotation factor analysis was performed to identify main factors that could represent the main sources of air pollution in Piracicaba (Weiss 1971), reducing the analysis to a small number of factors. Factor analysis is a multivariate technique that allows for the combination of multiple variables into few factors based on their degree of correlation, thereby reducing the number of elements included in the analysis. Using the varimax rotation technique, we identified three main factors from the 17 tracer elements mentioned above: biomass burning and soil dust (factor 1), industrial emissions (factor 2), and fuel or automotive emissions (factor 3) (Table 1). Additional models were built using the factors instead of tracer elements in single-and three-factor models.

Results were expressed in terms of percentage increases in respiratory hospital admissions for interquartile range (IQR) increases in air pollutant concentrations. To carry out the statistical analyses, we used S-Plus (version 4.536; Insightful Corporation, Seattle, WA, USA).

## Results

At the time of the study, 26% of the Piracicaba population were children and adolescents < 13 years of age, and 7% were > 64 years of age. There were 673 hospital admissions due to respiratory diseases among children and 275 among the elderly. Table 2 shows the daily descriptive analysis of child and elderly respiratory hospital admissions and temperature and humidity of the city of Piracicaba during the study period. The average number of child respiratory hospital admissions was more than twice that of the elderly, although the child population was almost four times the elderly population. The weather in Piracicaba is warm (climate characterized as subtropical Cw; humid with dry winter), and low temperatures are very rare.

Table 3 presents the descriptive analysis of the PM and its main elements measured during the entire period of study and burning and nonburning periods. Concentrations of PM_10_, PM_2.5_, and BC presented large seasonal variability with higher concentrations during the burning period compared with the nonburning period. For the elements generated mainly by biomass burning and soil dust, their concentrations increased from 2-fold to 4-fold between nonburning and burning periods. During the entire period of study, the PM_10_ concentration surpassed the standard limit adopted for this pollutant (50 μg/m^3^).

**Figure 2:**
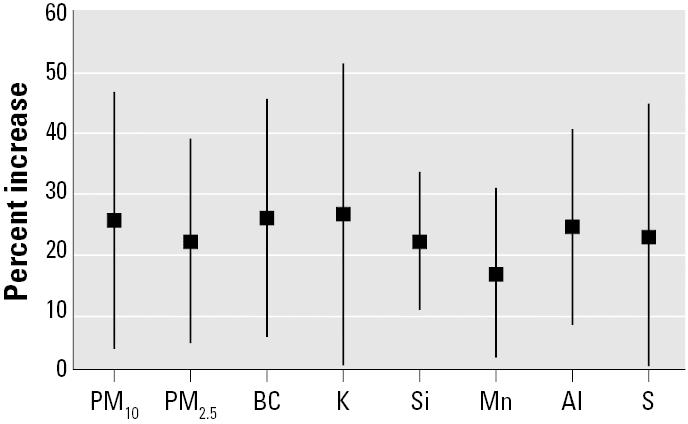
Percentage increases and 95% confidence intervals in child respiratory hospital admissions due to interquartile range increases in PM_10_, PM_2.5_, BC, Al, Si, Mn, K, and S during the period of study.

Daily variations in PM_10_ and PM_2.5_ as well as in BC, Al, Si, and K, which are products of biomass burning and soil dust (Lara et al. 2005), were significantly associated with child respiratory hospital admissions even after controlling for season and weather (Figure 2). Mn and S, proxies of industrial and automotive emissions, respectively, also presented effects on respiratory hospital admissions.

Furthermore, we observed associations between daily variations in PM_10_, BC, and K and elderly respiratory hospital admissions for the entire period (Table 4).

**Figure 3:**
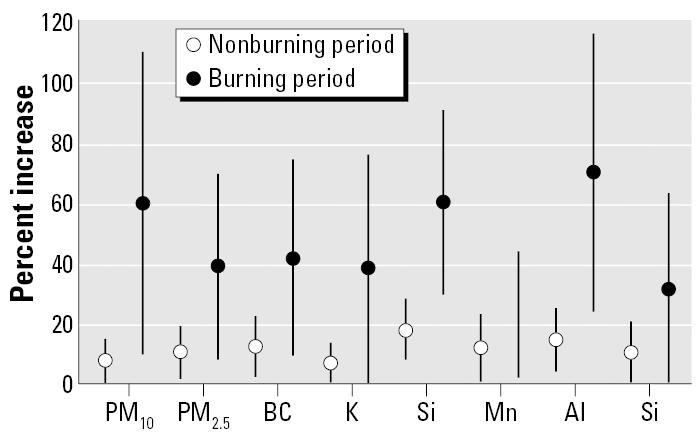
Percentage increases and 95% confidence intervals in child respiratory hospital admissions due to mean levels of PM_10_, PM_2.5_, BC, K, Si, Mn, Al, and S during burning and nonburning periods.

Figure 3 presents the estimated percentage increases in child respiratory hospital admissions shown by burning and nonburning periods. We observed that PM_2.5_, PM_10_, BC, and elements from PM_2.5_ generated from biomass burning and soil dust promoted higher effects during the burning period than during the nonburning period. Because the burning period is also the period with lower humidity and temperature, which impair air pollution dispersion, increases were observed in the elements generated from other sources, and consequently, higher respiratory effects resulted.

**Figure 4:**
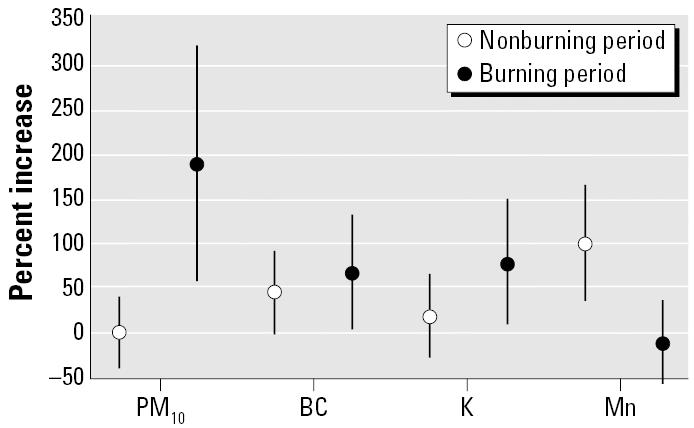
Percentage increases and 95% confidence intervals in elderly respiratory hospital admissions due to mean levels of PM_10_, BC, K, and Mn during burning and nonburning periods.

Among the elderly during the sugar cane–burning period (Figure 4), we also observed significant increases in respiratory hospital admissions due to increases in PM_10_, BC, and K, a proxy of biomass burning. Mn, a proxy of industrial emissions, presented significant associations with elderly hospital admissions during the nonburning period, showing a pattern of susceptibility different from those observed among children.

Models using factors (factor 1, biomass burning and soil dust; 2, industrial emissions; 3, automotive emissions) instead of pollutants and tracer elements were essential in specifying the source with the major impact on respiratory diseases among elderly and children. Factor 1 presented the highest effect on respiratory hospital admissions in both single- and three-factor models for children (Table 5) and the elderly (Table 6).

## Discussion

In contrast to large cities, such as São Paulo; Santiago, Chile; New York, New York; Baltimore, Maryland; and cities in Pakistan and China, where the main sources of aerosol particles are emissions linked to fossil fuel combustion (Artaxo et al. 1999; Castanho and Artaxo 2001; Conner et al. 2001; Lena et al. 2002; Zhang et al. 2002), the main source of aerosol in Piracicaba is biomass burning (Martinelli et al. 2002). In Piracicaba, sugar cane burning contributed 60% of the fine-mode aerosol mass. The second major source was resuspended soil dust, representing 14% of the PM_2.5_. Industry and oil combustion each contributed 12% of the total PM_2.5_ mass (Lara et al. 2005).

Studies have already shown that land use can influence the atmospheric composition in the Amazon Basin (Artaxo et al. 1999; Yamasoe et al. 2000) and in the State of São Paulo (Lara et al. 2001; Martinelli et al. 2002). Particularly, in the Piracicaba basin, the main land use is sugar cane plantations, which cover almost 80% of the total area.

In the present study, increases in PM_10_ and PM_2.5_ were strongly associated with respiratory hospital admissions. The magnitudes of those effects were slightly higher than that found in São Paulo, the largest Brazilian city (Braga et al. 1999, 2001; Lin et al. 1999). When the magnitude of the effects of PM_10_ and PM_2.5_ were assessed for the same end point, there were no differences between them. This could be explained by the fact that we worked with total PM_10_ measurements instead of just the coarse-mode fraction. Hence, it is possible to assume that the effects observed for PM_10_ include PM_2.5_ effects.

However, in a region with at least three important sources of PM air pollution—biomass burning and industrial and automotive emissions—it is necessary to identify the air pollution source responsible for most of the respiratory hospital admissions. In this context, the association of elemental components of PM_2.5_ that are generated mainly from biomass burning and soil dust (BC, Al, Si, and K) and the results of factor analysis showing that factor 1 (biomass burning) was the most associated with respiratory hospital admissions reinforce the role of this source of air pollution on child and elderly respiratory diseases in this region, despite the contribution of industrial and automotive emissions.

The use of factor analysis to identify and apportion ambient concentrations to sources has been used in the past, and a workshop sponsored by the U.S. Environmental Protection Agency explored the use of resolved source contributions in health effects models (Thurston et al. 2005). For this workshop, multiple groups of investigators analyzed PM composition data sets from two U.S. cities, and although different factor analysis methods were used, similar source profiles were extracted from these sets, with a good agreement among the major resolved source types. The same investigators also found similar health effects associations (Hopke et al. 2005; Ito et al. 2005; Laden et al. 2000; Mar et al. 2005). In our data set, we had 1 year of daily data, which is less than that in the studies described above; the factor analysis chose only some factors, and in the model we adjusted for all potential confounders that have been shown to be the most relevant. The associations of health effects with the metals are still under investigation, and although some associations might be due to chance, evidence for them has been found in the past. We believe that our results are not due to chance, even though other studies are needed to confirm these findings.

Studies have shown that population exposure to high levels of pollutants can increase the risk of acute respiratory infections, chronic obstructive pulmonary disease, and lung cancer (Smith et al. 1999). Children are the most susceptible group. Studies performed in the city of São Paulo have shown adverse effects of PM on inflammatory and infectious diseases of both upper and lower respiratory tracts (Farhat et al. 2005; Lin et al. 1999). Indeed, acute lower respiratory infections are the single most important cause of mortality in children > 5 years of age (Bruce et al. 2000). Globally, about 43% of the total burden of disease due to environmental risks falls on children > 5 years of age, although they make up only 12% of the population (Smith et al. 1999).

This study adopted total respiratory hospital admissions of children and elderly instead of specific-respiratory disease diagnoses because of the small number of events observed in the city during the study period. Moreover, this approach avoided misclassification of disease-specific diagnoses.

Around Piracicaba, the percentage increase in child respiratory hospital admissions in relation to the mean levels of PM_10_, PM_2.5_, BC, Al, Si, Mn, K, and S was two to three times higher in the burning season than in the nonburning season, compromising the health of this population. Among the elderly, the same pattern of effects was observed between burning and nonburning periods. It undoubtedly shows that the months of the year when sugar cane is burned are the most dangerous for the inhabitants of the region.

In fact, the association between exposure to indoor and outdoor biomass smoke and health effects has already been reported in some areas of Asia and India (Behera et al. 1994; Phonboon et al. 1999). Deposition of carbon in the lungs occurred consistently in patients exposed to biomass burning (Bruce et al. 2000). However, different from most of the mentioned regions where outdoor biomass burning events are episodic, biomass burning is a common and scheduled activity in Piracicaba and other Brazilian sugar cane plantation areas, exposing the population on a regular basis.

The mechanisms whereby PM causes airway diseases have been studied. Oxidative stress, caused by the release of oxidant radicals by inflammatory cells, may be an important component in promoting inflammation and respiratory cell damage, compromising pulmonary function, increasing susceptibilities to allergens, and increasing the incidence of respiratory infections (Bernstein et al. 2004; Bruce et al. 2000).

The population of these areas of Brazil has been exposed to sugar cane burning for at least 6 months every year for the last four decades. The health effect is determined not just by acute exposures to high pollution levels but also, and more importantly, by the length of time that people spend breathing polluted air chronically. Similar to this study, the acute effect of sugar-cane–generated air pollution was assessed previously in São Paulo State (Arbex et al. 2000). However, the magnitude of chronic effects is not known.

Alcohol produced from sugar cane is a renewable fuel and is less pollutant than fossil fuel. Brazil is the largest producer of sugar and alcohol from sugar cane in the world. Plantations cover about 5 million hectares and surround a great number of cities, mainly in the southeastern region of the country. About 80% of the sugar cane crops are burned to facilitate harvesting every year (Brazilian Agriculture Ministry 2005).

The addition of alcohol to gasoline and national policies to reduce automotive emissions contributed to a decrease in air pollution in Brazilian urban centers in the last 20 years. Nowadays, the use of alcohol as automotive fuel is under study or has already been partially adopted in many countries. Hence, it is clear that ethanol production will tend to grow in this century. To avoid secondary health damages, the procedure of burning the crops before harvesting should be banished.

In summary, we showed that air pollution from biomass burning causes damage to the respiratory system, leading to an increase in respiratory hospital admissions. This effect is higher for children and the elderly, and it is similar to that observed in urban areas due to exposure to industrial and vehicle-emitted air pollutants. Finally, it is necessary to keep in mind the close relation between health effects and air pollution. This concern should be included in public efforts for sustainability and improving environmental health conditions.

## Figures and Tables

**Table 1:** Factor analysis using varimax rotation for PM_2.5_ tracer elements.

	Factor (main sources)			
Tracer elements of PM_2.5_	Biomass burning	Industrial	Automotive	Communalities
Si	0.876	0.221	0.382	0.911
S	0.509	0.090	0.856	0.889
Cl	0.475	0.051	0.726	0.763
K	0.888	−0.045	0.385	0.898
V	0.295	0.288	0.665	0.973
Fe	0.619	0.671	0.377	0.874
Ni	0.283	0.317	0.686	0.982
Cu	0.256	0.678	0.200	0.910
Zn	0.039	0.836	0.086	0.879
Br	0.610	0.415	0.238	0.813
Pb	−0.089	0.640	−0.004	0.913
Percent variance	59	21	10	

The communality expressed for each variable represents the fraction of the respective variable that is explained by the retained factors. In this case, the communalities were typically higher than 82% (PM_2.5_). This indicated that the factors could explain most of the data variability.

**Table 2:** Descriptive analyses of child and elderly respiratory hospital admissions, temperature, and humidity of Piracicaba during the study period.

	Daily mean	SD	Minimum	IQR	Maximum	*n* (days)
Hospital admissions						
Children	2.2	1.7	0.0	2.0	8.0	306
Elderly	0.9	1.0	0.0	1.0	5.0	306
Weather						
Minimum temperature (°C)	15.8	4.1	5.5	6.5	23.2	298
Relative humidity (%)	81.7	9.5	52.0	12.0	100.0	298

**Table 3:** Descriptive analysis of PM_10_, PM_2.5_, BC, Al, Si, S, K, and Mn in the entire study period and during burning and nonburning seasons.

	Entire period		Burning period		Nonburning period	
Pollutant	Mean ± SD	IQR	Mean ± SD	IQR	Mean ± SD	IQR
PM_10_ (μg/m^3^)	56.1 ± 49.8	42.9	87.7 ± 57.9	89.5	28.9 ± 12.8	15.0
PM_2.5_ (μg/m^3^)	16.1 ± 12.4	10.2	22.8 ± 14.7	17.3	10.0 ± 4.6	5.5
BC (μg/m^3^)	2.1 ± 2.0	1.9	4.2 ± 2.3	2.9	1.8 ± 0.7	1.0
Al (ng/m^3^)	166.3 ± 260.7	193.7	370.8 ± 317.5	480.1	157.9 ± 149.7	124.9
Si (ng/m^3^)	404.5 ± 369.1	275.7	545.3 ± 462.9	669.2	283.9 ± 201.8	234.6
S (ng/m^3^)	1362.1 ± 1,049.2	1009.6	1922.9 ± 1,237.5	1370.5	881.4 ± 497.2	492.7
K (ng/m^3^)	380.2 ± 359.0	383.5	626.6 ± 390.4	539.1	168.9 ± 113.4	114.2
Mn (ng/m^3^)	12.6 ± 10.0	9.0	16.9 ± 12.4	12.3	8.8 ± 4.6	6.82

**Table 4:** Percentage increases and 95% confidence intervals (CIs) in elderly respiratory hospital admissions due to interquartile range increases in PM_10_, BC, and K during the period of study.

Pollutant	Percentage increase (95% CI)
PM_10_	31.03 (1.25–60.81)
BC	36.41 (11.14–61.68)
K	46.74 (11.67–81.82)

CI, confidence interval.

**Table 5:** Regression coefficients, SEs, and statistical significance of the models for child respiratory hospital admissions using single or three factors as independent variables.

	Model					
	Single factor			Three factors		
	β	SE	*p*-Value	β	SE	*p*-Value
Biomass burning	0.1996	0.1138	0.0867	0.2138	0.1180	0.0775
Industrial	0.0722	0.0922	0.4378	0.0559	0.0921	0.5470
Automotive	0.0426	0.0949	0.6559	0.0832	0.0935	0.3791

**Table 6:** Regression coefficients, SEs, and statistical significance of the models for elderly respiratory hospital admissions using single or three factors as independent variables.

	Model					
	Single factor			Three factors		
	β	SE	*p*-Value	β	SE	*p*-Value
Biomass burning	0.4156	0.1522	0.0092	0.3527	0.1644	0.0380
Industrial	−0.0990	0.1380	0.4771	−0.0703	0.1356	0.6070
Automotive	−0.3009	0.1767	0.0961	−0.1753	0.1541	0.2622
